# Acute Manipulation of Diacylglycerol Reveals Roles in Nuclear Envelope Assembly & Endoplasmic Reticulum Morphology

**DOI:** 10.1371/journal.pone.0051150

**Published:** 2012-12-05

**Authors:** Marie-Charlotte Domart, Tina M. C. Hobday, Christopher J. Peddie, Gary H. C. Chung, Alan Wang, Karen Yeh, Nirmal Jethwa, Qifeng Zhang, Michael J. O. Wakelam, Rudiger Woscholski, Richard D. Byrne, Lucy M. Collinson, Dominic L. Poccia, Banafshé Larijani

**Affiliations:** 1 Cell Biophysics Laboratory, London Research Institute, Cancer Research United Kingdom, London, United Kingdom; 2 Electron Microscopy Unit, London Research Institute, Cancer Research United Kingdom, London, United Kingdom; 3 Department of Biology, Amherst College, Amherst, Massachusetts, United States of America; 4 The Babraham Institute, Babraham Research Campus, Cambridge, United Kingdom; 5 Department of Chemistry, Faculty of Natural Sciences and Institute of Chemical Biology, Imperial College London, London, United Kingdom; Cinvestav-IPN, Mexico

## Abstract

The functions and morphology of cellular membranes are intimately related and depend not only on their protein content but also on the repertoire of lipids that comprise them. In the absence of *in vivo* data on lipid asymmetry in endomembranes, it has been argued that motors, scaffolding proteins or integral membrane proteins rather than non-lamellar bilayer lipids such as diacylglycerol (DAG), are responsible for shaping of organelles, local membrane curvature and fusion. The effects of direct alteration of levels of such lipids remain predominantly uninvestigated. Diacylglycerol (DAG) is a well documented second messenger. Here we demonstrate two additional conserved functions of DAG: a structural role in organelle morphology, and a role in localised extreme membrane curvature required for fusion for which proteins alone are insufficient. Acute and inducible DAG depletion results in failure of the nuclear envelope (NE) to reform at mitosis and reorganisation of the ER into multi-lamellar sheets as revealed by correlative light and electron microscopy and 3D reconstructions. Remarkably, depleted cells divide without a complete NE, and unless rescued by 1,2 or 1,3 DAG soon die. Attenuation of DAG levels by enzyme microinjection into echinoderm eggs and embryos also results in alterations of ER morphology and nuclear membrane fusion. Our findings demonstrate that DAG is an *in vivo* modulator of organelle morphology in mammalian and echinoderm cells, indicating a fundamental role conserved across the deuterostome superphylum.

## Introduction

Although membrane morphology and function depend on associated proteins, events such as fusion and fission or organelle shaping involve asymmetric alterations of lipid composition across the membrane. Biophysical studies show that fusion of protein-free lipid bilayers is exquisitely sensitive to lipid composition.

Theoretical studies indicate that localised membrane morphology is dependent on the chemical structure of the lipids comprising a monolayer. Local morphology is determined by spontaneous curvature. Lipids with positive spontaneous curvature (e.g. polyphosphoinositides) favour bending away from the head groups and those with negative spontaneous curvature (e.g. diacylglycerol [DAG]) favour bending towards the head groups [1], [2]. Lipids promoting negative curvature facilitate fusion [2], [3], [4] and favour highly curved regions seen in membrane tubules or small vesicles [5], [6], [7].

Roles for DAG in initiating fusion of biological membranes including NE precursors have been demonstrated in cell-free assays [8], [9], [10], [11]. In cells, a lipin family of lipid phosphatases converts phosphatidic acid (PtdOH) to DAG. Deletion of the lipin gene in yeast and down-regulation of lipin by RNAi in *C. elegans* result in irregular nuclei with expanded NEs [12] and NE disruption [13], but neither study directly manipulated the lipid.

The 1,2 DAG isomer of DAG plays a well documented role as a second messenger in signalling pathways, whereas the 1,3 isomer is not a second messenger but has the same physical properties [14]. 1,3 DAG therefore can be used for studying the physical effects of DAG on membrane dynamics and morphology [15].

In the absence of *in vivo* data on the role of lipid asymmetry in endomembrane morphology, it has been argued that motors, scaffolding proteins or integral membrane proteins rather than non-lamellar bilayer lipids are responsible for shaping of organelles, local membrane curvature and fusion [16], [17]. Investigation of the contribution of non-lamellar lipids *in vivo* has been hampered by the difficulty of rapidly altering composition in specific subcellular compartments. To examine the *in vivo* participation of DAG in NE (a subdomain of the endoplasmic reticulum [ER]) formation and in ER architecture, we used a tool that can recruit lipid-modifying enzymes to specific endomembranes to rapidly deplete DAG.

By targeting DAG kinase (DGK) or SKIP phosphatase [18] to specific membranes we showed dose-dependent major alterations in ER morphology (curvature) and ability to complete NE formation. We also acutely attenuated DAG levels by microinjection of DGK or Syn1 phosphatase into echinoderm eggs which resulted in blocks or delays in nuclear membrane fusion and reorganised ER from tubules to densely packed multi-lamellar sheets. Our data illustrate a critical role for DAG in nuclear membrane assembly and fusion and in the maintenance of ER architecture, which we interpret as dependent on lipid composition effects on membrane curvature not due solely to proteins. Our findings also imply conserved mechanisms operating from embryonic to differentiated cells across the deuterostome superphylum.

## Results and Discussion

Theoretical considerations suggest that membrane curvature and bending energy are fundamental to the shaping of organelles and to fusion and fission events [19]. *In vivo*, the energetic costs for maintaining organelle shape or highly localised membrane curvature have been ascribed to a wide variety of proteins including scaffolds, motors and wedges in order to stabilise curved structures like ER tubules and small vesicles or facilitate localised curvature at fusion stalks [2], [16], [20], [21], [22], [23]. Here we directly test the role of lipids in ER morphology and NE formation in mammalian cells, echinoderm embryos and unfertilised oocytes by acutely depleting *in vivo* the neutral lipid DAG.

### Localisation of DAG in live mammalian cells

**Figure 1:**
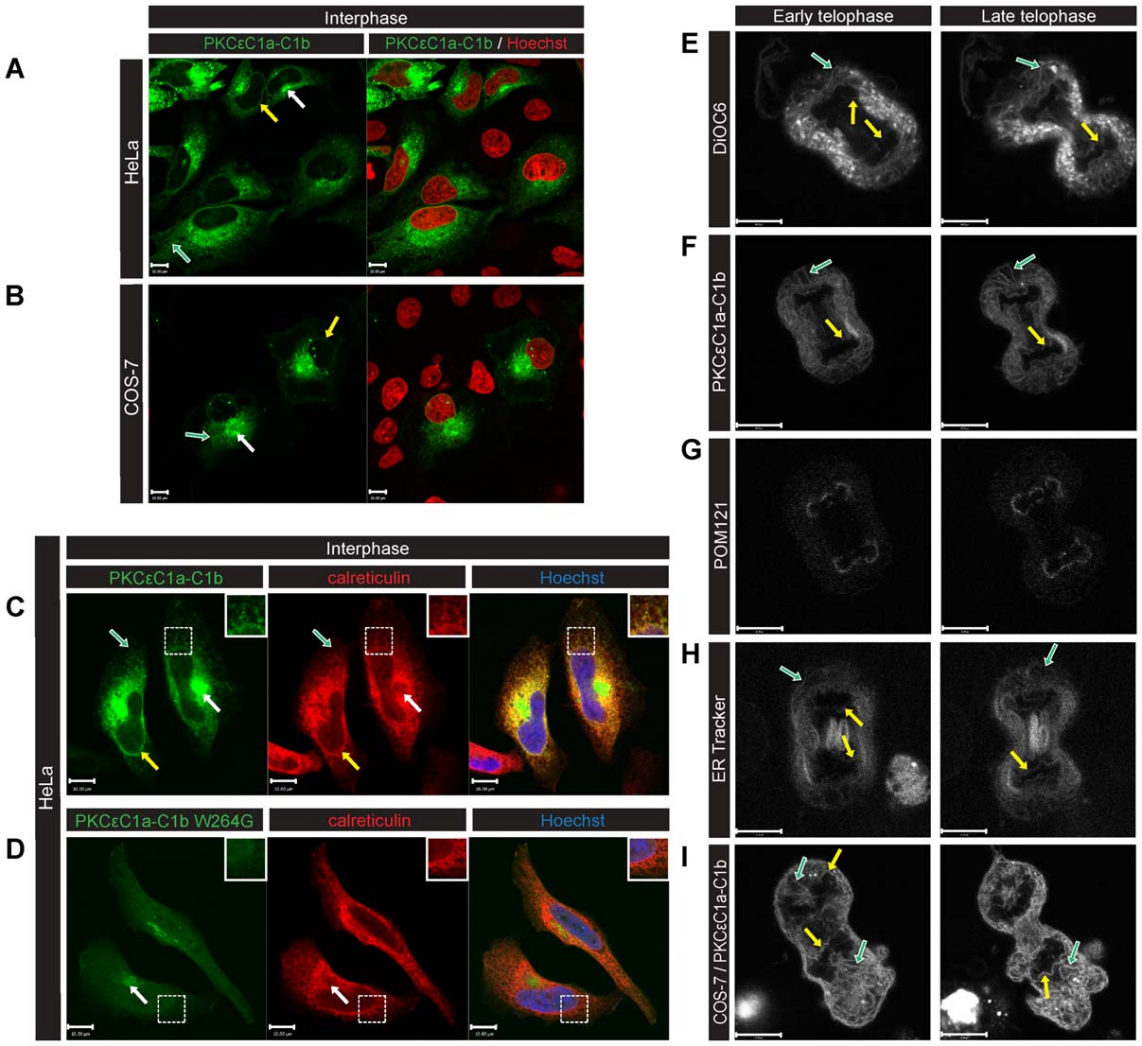
Diacylglycerol localises to the NE, Golgi and ER in mammalian cells. HeLa (A) and COS-7 (B) cells were transfected with EGFP-PKCεC1aC1b (DAG probe-green), fixed and imaged by confocal microscopy. DAG was localised at the NE (yellow arrows), ER (green arrow) and Golgi (white arrows). (C–D) Calreticulin (ER marker) was detected by indirect immunofluorescence (red). Apart from a minor detection of the Golgi, the signal at the ER and NE (insets) was absent in cells transfected with the DAG non-binding mutant (C1b W264G) (D). HeLa (E-H) and COS-7 (I) cells were followed through mitosis by live confocal microscopy. EGFP-PKCεC1aC1b in HeLa (F) and COS-7 (I) presented similar distributions as DiOC_6_ (E), GFP-POM121 (G), and ER tracker (H). ER tubules (green arrows) and NE reformation (yellow arrows) were observed. To label chromatin, cells were incubated with Hoechst 333432 or transfected with mCherry-H2B. Scale bars: 10 μm.

C1 domains are established probes for DAG localisation [24]. To detect the distribution of DAG in mammalian cells, we constructed an EGFP-C1a-C1b probe from PKCε. Fig. 1A shows strong signals from this probe in interphase HeLa cells at the Golgi (white arrow), ER (green arrow) and NE (yellow arrow) compared to the plasma membrane. Identical localisation of DAG was observed in COS-7 cells (Fig. 1B). The probe co-localised with the ER network, marked with calreticulin (Fig. 1C). To verify that detection was specific, we mutated the tryptophan 264 residue of PKCε-C1b to glycine. The wild type domain confers binding to DAG with an affinity of 52 nM [25]; the mutant has a significantly lower affinity. Apart from a minor signal at the Golgi, ER and NE localisation of the EGFP-C1a-C1b was abolished with the mutant (W264G) probe (Fig. 1D). More than 90% of the cells transfected with the mutant probe lost ER and NE signal. Furthermore, interphase cells labelled with NBD-DAG confirmed that DAG localises to the Golgi, ER and NE (Fig. S1A). To examine if the probe functioned correctly we tested the translocation of the probes to the plasma membrane in phorbol ester (PMA)-treated cells. Upon treatment the wild type C1 domain signal translocated to the plasma membrane but the W264G mutant did not respond (Fig. S1B).

The NE breaks down at prometaphase and starts reforming at anaphase. Fig. 1 shows the localisation of DiOC_6_, [general membrane marker] (Fig. 1E) [26], the DAG probe (Fig. 1F and Movie S1) and GFP-POM121 [nuclear pore marker] (Fig. 1G) from early to late telophase in HeLa cells. The portion of the envelope facing the mid-body was relatively free of pores. Fig. 1H shows the redistribution of ER during mitosis. The results demonstrate that DAG was present in reforming nuclear membranes. Using the same probe, identical localisation of DAG was observed in COS-7 cells (Fig. 1I).

### NE formation and ER morphology depend on DAG

The rapalogue dimerisation device was pioneered in our laboratory for the acute and inducible depletion of phosphatidylinositol(3)phosphate (PtdIns3P) from early endosomes [27]. We used this device to specifically target enzymes that prevent the accumulation of DAG to the ER and its NE subdomain. Lamin B receptor (LBR) was used to target these compartments. LBR, an inner nuclear membrane protein facing the nucleoplasm [28], [29] also localised to the ER in interphase cells (Fig. S2A) and translocated to the ER during mitosis. The truncated transmembrane domain (LBRΔTM2–8) and full length LBR behaved in the same manner (Fig. S2B).

To deplete DAG, we used DAG kinase ε (DGKε) or SKIP (phosphoinositide 5-phosphatase). DGKε phosphorylates DAG to PtdOH and SKIP dephosphorylates phosphatidylinositol(4,5)bisphosphate (PtdIns(4,5)P_2_) to phosphatidylinositol(4)phosphate (PtdIns4P), reducing the physiological substrate for phosphatidylinositol-specific phospholipase C. Endogenous PtdIns(4,5)P_2_ is present at the ER and perinuclear regions as shown by Watt *et al.* [30] and in Fig. S3; therefore recruitment of SKIP to the nuclear membrane will dephosphorylate PtdIns(4,5)P_2_ to PtdIns4P. DGKε selectively phosphorylates unsaturated DAG species [31]. To show that unsaturated DAG species were present, we isolated nuclei enriched in ER and NE membrane and extracted their lipids. Using liquid chromatography tandem mass spectrometry we determined that DAG was composed predominantly of unsaturated fatty acid species (particularly 34:1-DAG) (Methods and Fig. S2C).

**Figure 2:**
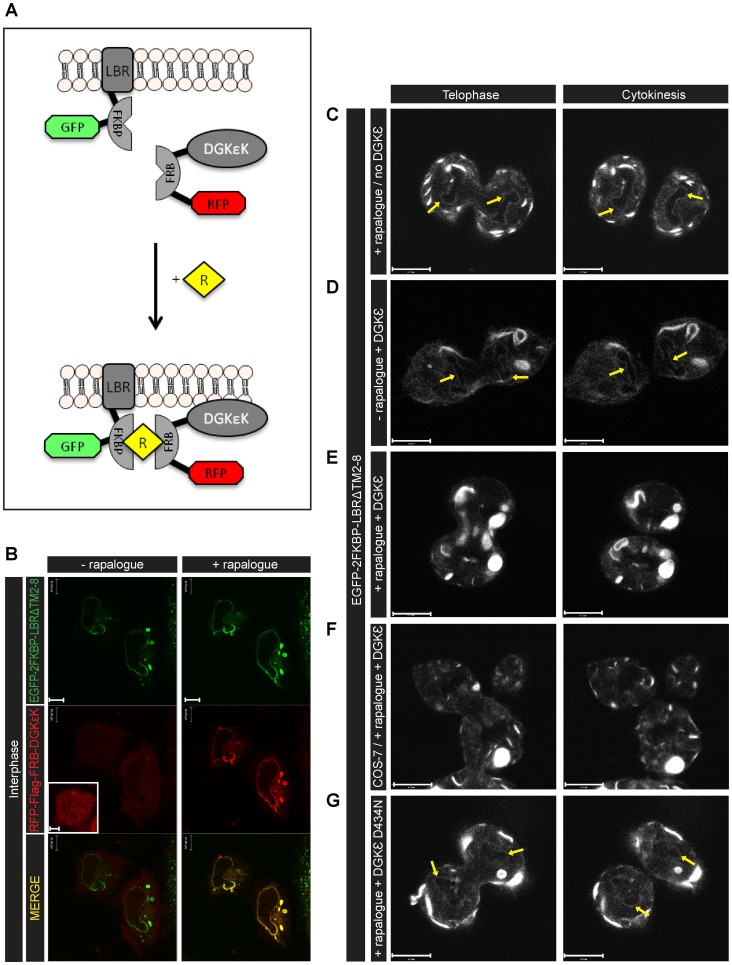
Acute depletion of DAG results in an incomplete NE. (A) Diagram of the rapalogue dimerisation device. After rapalogue (R) treatment, RFP-Flag-FRB-DGKεK (DGKε) dimerises with EGFP-2FKBP-LBRΔTM2-8 (LBR) and is recruited to LBR in the ER and NE. (B) Confocal images of live interphase HeLa cells transfected with LBR (green) and DGKε (red) show the recruitment of DGKε (inset) to the NE and ER, 45 min after addition of 500 nM rapalogue. (C) HeLa cells transfected with EGFP-2FKBP-LBRΔTM2-8 (LBR) only and treated with rapalogue showed a normal NE reformation (yellow arrows) between late telophase (left panel) and cytokinesis (right panel), similarly to what was observed in (D) LBR and DGKε co-expressing HeLa cells in the absence of rapalogue. In HeLa (E) and COS-7 (F) cells treated with rapalogue NE reformation was impaired. Images representative of n = 10 experiments. (G) When DGKε was replaced by its inactive mutant (D434N) the NE formation was normal (yellow arrows). Images representative of n = 6 experiments. Scale bars: 10 μm.

Fig. 2A depicts the rapalogue dimerisation device. At interphase, RFP-Flag-FRB-DGKε (DGKε) [or RFP-Flag-FRB-SKIP (SKIP)] colocalises with EGFP-2FKBP-LBRΔTM2-8 (LBR) in the ER and NE upon rapalogue-triggered dimerisation (Fig. 2B). At high LBR expression levels, regions of intense fluorescence were observed in the cytoplasm, which may be indicative of alterations of ER morphology.

Localisation of LBR during mitosis is illustrated in Fig. 2C-G. Rapalogue alone without DGKε did not affect the formation of the NE (Fig. 2C). Without rapalogue and with DGKε, NE (yellow arrows) assembly was complete at late telophase (Fig. 2D and Movie S2). However, with rapalogue at high levels of DGKε, depletion of DAG resulted in NE malformation and a prominent enhancement of regions of intense fluorescence (Fig. 2E and Movie S3). Remarkably, both HeLa and COS-7 (Fig. 2F) cells divided with an incomplete NE. Nuclear membranes were formed normally when the inactive kinase DGKε (D434N) was used (Fig. 2G). Therefore, when DAG was not phosphorylated to PtdOH the nuclear membrane formed normally.

**Figure 3:**
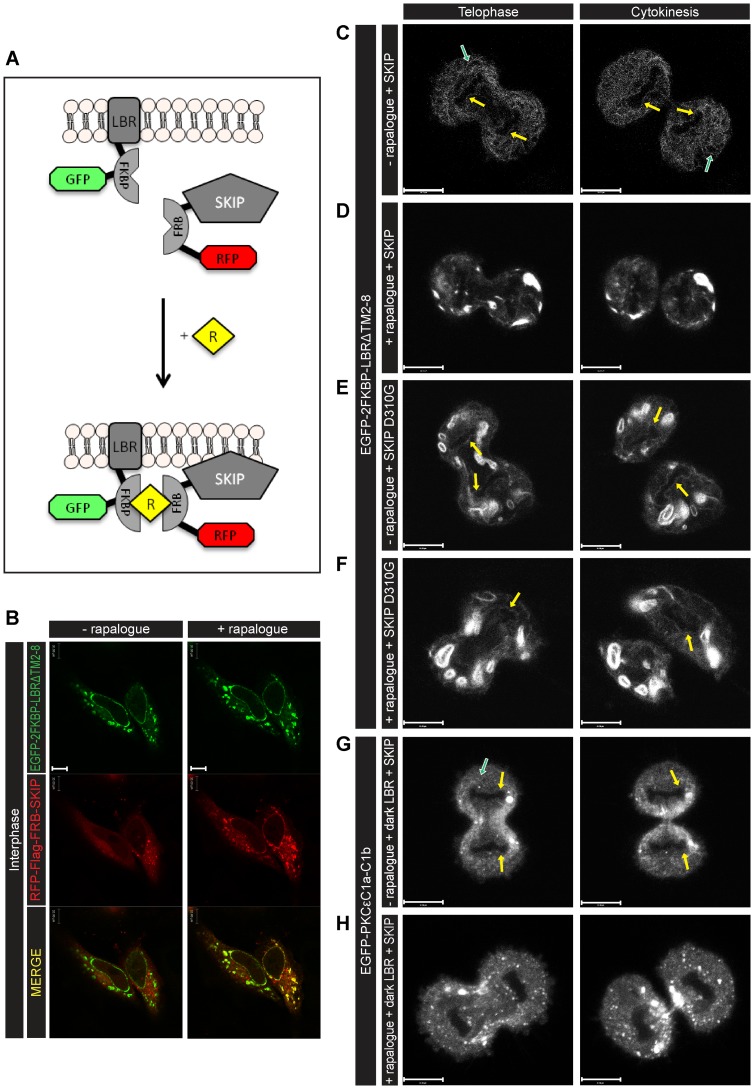
Acute depletion of PtdIns(4,5)P_2_ results in an incomplete NE: DAG is not observed upon malformation of the NE. (A) Diagram of the rapalogue dimerisation device. After rapalogue (R) treatment, RFP-Flag-FRB-SKIP (SKIP) dimerises with EGFP-2FKBP-LBRΔTM2-8 (LBR) and is recruited to LBR in the ER and NE. (B) Confocal images of live interphase HeLa cells transfected with LBR (green) and SKIP (red) show the recruitment of SKIP to the NE and ER, 45min after addition of 500 nM rapalogue. (C) LBR localisation during mitosis in LBR and SKIP co-expressing HeLa cells in the absence of rapalogue shows complete NE reformation (yellow arrows) between telophase (left panel) and cytokinesis (right panel). (D) In HeLa cells treated with rapalogue NE reformation was impaired. (E–F) When SKIP was replaced by its inactive mutant (D310G) the NE formation was normal (yellow arrows), in the absence (E) or presence (F) of rapalogue. (G-H) EGFP-C1a-C1b (DAG probe-green) localisation during mitosis in dark (EGFP) LBR and SKIP co-expressing cells. In the absence of rapalogue (G), NE formation was normal (yellow arrows) and ER tubules were visible (green arrow), contrary to what was observed in the presence of rapalogue (H). Images representative of n = 3 experiments. Scale bars: 10 μm.

To ensure that incomplete NE and altered ER phenotypes were due to acute depletion of DAG rather than increased PtdOH, PtdIns(4,5)P_2_, the precursor of DAG, was converted by dephosphorylation to PtdIns4P by recruiting SKIP to LBR. Fig. 3A shows the rapalogue dimerisation device with SKIP. The recruitment of SKIP to LBR at interphase is shown in Fig. 3B. NE formation was normal during mitosis when LBR and SKIP were co-expressed in HeLa cells (Fig. 3C), but with rapalogue the NE was incomplete and increased cytoplasmic fluorescence occurred (Fig. 3D). The nuclear membrane was formed normally when the phosphatase inactive mutant (D310G) was used, either in the absence (Fig. 3E) or presence (Fig. 3F) of rapalogue. These phenotypes were similar to the DGKε experiments.

To *directly* observe the modification of DAG during mitosis, HeLa cells were transfected with dark-EGFP-2FKBP-LBRΔTM2-8, SKIP and EGFP-C1a-C1b. Fig. 3G shows that EGFP-C1a-C1b localised to the nuclear envelope (yellow arrows) and the ER (green arrows) without rapalogue. However, in the presence of rapalogue, the nuclear envelope was not formed and the DAG probe did not localise to the nuclear membrane (Fig. 3H). These experiments show that DAG did not accumulate at the nuclear envelope upon the modification of its precursor.

Cell cycle synchronising reagents were not used in any of our experiments as these induce various artefacts [32]. Therefore all rapalogue experiments were single cell experiments performed between 6 to 11 times with 76–95% reproducibility (Methods and **Table** **1**). Since in the rapalogue experiments DAG levels were acutely modified only at the ER and NE, membrane lipid composition was rapidly altered and other subcellular compartments were not directly affected. Therefore unlike with knockdowns or mutations of proteins, where the cell membranes have time to adjust to a new steady state, the effects on the targeted subcellular compartments were rapid and targeted.

**Table 1:** Quantification of NE phenotype in single cell experiments of HeLa cells co-expressing LBR and DGKε.

Condition	Number of independent experiments	Number of mitotic LBR/DGK-expressing cells	Number (%)of cells with complete NE at cytokinesis	Number/% of cells with fragmented NE at cytokinesis
LBR/DGK WT + rapalogue	10	25	6 (24%)	19 (**76%**)
LBR/DGK D434N + rapalogue	6	19	18 (**94.7%**)	1 (5.3%)
LBR/DGK WT + rapalogue + DAG-containing SUVs (*Rescue*)	11	58	52 (**89.7%**)	6 (10.3%)

Treatment with rapalogue shows that 76% of cells co-expressing LBR and DGKε at cytokinesis had an incomplete NE, the mutant DGKε shows that 94.7% of the cells had a complete NE. The DAG-containing SUV rescue experiments show 89.7% of the cells had a complete NE at cytokinesis.

### Ultrastructure of altered NE and ER membranes revealed by correlative light and electron microscopy

Correlative light and electron microscopy (CLEM) was used to determine the structure of the fragmented NE and to assess whether enhanced cytoplasmic fluorescence signals indicated defects in ER morphology. CLEM workflow is outlined in Fig. S4. Live HeLa cells were imaged in the confocal at the required mitotic stage, then fixed and embedded for transmission electron microscopy. Serial sections (70–80 nm) were collected and imaged, so that the entire structure of the nucleus and associated ER could be assessed [33]. Images were aligned, and ER and NE manually traced (segmented) and rendered into 3D models. A high level of precision is required for manual segmentation, each cell taking up to four weeks from initial fluorescence imaging to nanometre-scale 3D model generation. Therefore, CLEM experiments were performed once per condition and the quantitative analyses were provided by the confocal studies (Table 1).

**Figure 4:**
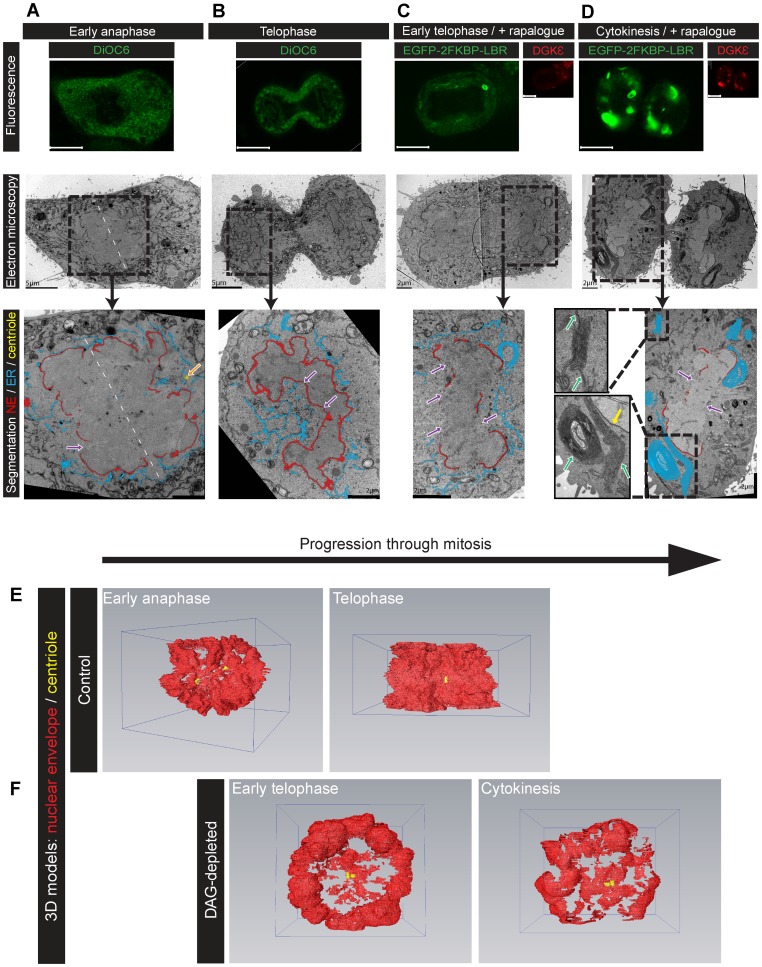
NE reformation is disrupted in a dose-dependent manner in DAG-depleted mitotic cells. (A–B) HeLa cells labelled with DiOC_6_ (green) were followed through mitosis by confocal microscopy, fixed at early anaphase (A) or telophase (B) and prepared for high-resolution imaging using CLEM (Fig. S4). The segmentation showed that at early anaphase the NE (red) was incomplete with wide gaps of 4 to 5 μm (purple arrows), at telophase the NE was close to completion with gaps of 50 nm. Segmentation of the ER (blue) at anaphase showed that it was mainly tubular. Note that the NE of the early anaphase cell was segmented around both sets of chromosomes. Dashed white line indicates axis of symmetry. Orange arrow highlights centriole (only one in this section). Serial sections are shown in Movie S4. (C-D) Same experiment with rapalogue-treated HeLa cells expressing LBR (green) and low (C) or high (D) levels of DGKε (red) fixed at early telophase (C) and cytokinesis (D). Dose-dependent effects upon DAG depletion included large gaps in the NE (purple arrows) and aggregation of the ER (blue). The ER phenotype consisted of large multi-lamellar sheets of membrane (insets-green arrows) with minimal NE contact (inset-yellow arrow). Movies of serial sections are shown in Movies S5–6. (E-F) 3D models reconstructed from manually-segmented CLEM serial images of control (E) and DAG-depleted (F) HeLa cells (Movie S7). In control cells, the NE at anaphase (red) was incomplete, while virtually complete at telophase. NE of the early anaphase cell was segmented around both sets of chromosomes. In DAG-depleted cells, the NE was not formed. Centrioles shown in yellow. Scale bars: fluorescence 10 μm; CLEM as indicated on the images.

Fig. 4A, B show controls labelled with DiOC_6_ fixed at anaphase (Fig. 4A) or telophase (Fig. 4B). At anaphase, the NE was incomplete with wide gaps of ∼4 to 5 μm (Fig. 4A-purple arrow) while at telophase the NE was close to completion with gaps of ∼50 nm (Fig. 4B-purple arrows and Movie S4). A 3D model of anaphase ER (blue) showed that it was mainly tubular whereas the telophase ER was a combination of sheets and tubules and the NE (red) was virtually complete. Since the positions of both sheets and tubules were correlated with confocal images of the live cells, these could not be attributed to fixation artefacts.

Fig. 4C shows a cell treated with rapalogue expressing low levels of DGKε. Chromosomal segregation had occurred at telophase but the NE resembled that of anaphase controls with large gaps of 2–5 μm (Movies S5). The gaps facing the mitotic poles were ∼5 μm and the membrane facing the mid-body was not formed. Cells treated with rapalogue expressing higher levels of DGKε (Fig. 4D) and hence more extensively depleted of DAG, had a highly fragmented NE and an aberrant ER morphology at cytokinesis (Movie S6). Segmentation showed several regions of ER consisting of large multi-lamellar sheets (Fig. 4D and Movie S7). Only a few ER tubules extended from the sheets to the fragmented NE (insets-green arrows). Therefore the intense fluorescence signal in confocal microscopy corresponded to dense aberrant multi-lamellar sheets.

Fig. 4E illustrates a 3D reconstruction of the NE at telophase. To our knowledge, the entire NE has not been previously reconstructed. Reconstruction from rapalogue-treated cells co-expressing LBR and DGKε shows clearly that with localised depletion of DAG the NE was not completely formed at cytokinesis (Fig. 4F and Movie S7).

### 1,3-DAG rescues the fragmented NE phenotype

**Figure 5:**
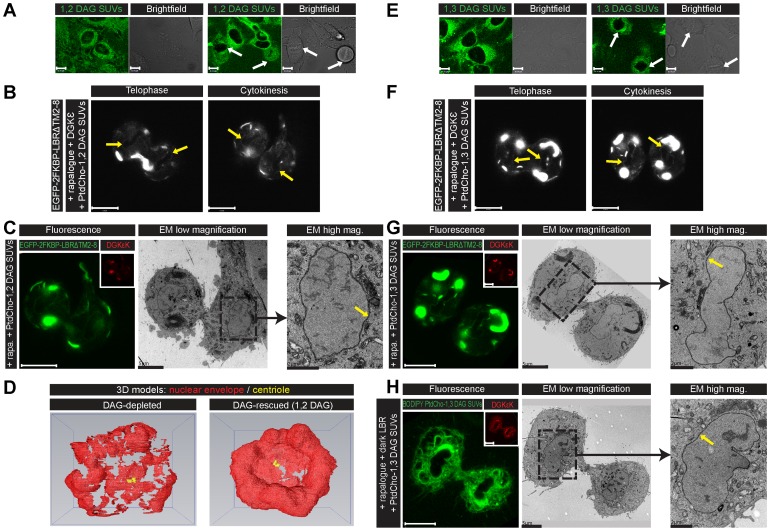
1,2- and 1,3-DAG rescue the fragmented NE phenotype. (A) Confocal images of live HeLa cells 1 min after addition of small unilamellar vesicles (SUVs) containing BODIPY-PtdCho and unsaturated 1,2 DAG (80∶20 mole% respectively). Incorporation of SUVs (green) into interphase and metaphase (white arrows) cells are shown. (B) LBR localisation during mitosis in rapalogue-treated, LBR and DGKε-expressing HeLa cells after addition at metaphase of SUVs containing PtdCho and unsaturated 1,2 DAG (80∶20 mole%). NE reformation (yellow arrows) was rescued. (C) Ultrastructure of the NE (yellow arrow) of the same cell at cytokinesis imaged using CLEM (Fig. S4). LBR localisation in green, DGKε in red. Serial sections are shown in Movie S8. (D) Comparison of 3D models reconstructed from serial images of DAG-depleted (left panel) and DAG-rescued (right panel) cells shows the NE reformed in the presence of 1,2 DAG. (E–G) Similar results as in (A–C) respectively were obtained with SUVs of the non-C1 domain-binding DAG isomer 1,3 DAG. (H) CLEM images of a rapalogue-treated, dark LBR and DGKε-expressing HeLa cell fixed at cytokinesis, after addition of (60∶40 mole %) SUVs with BODIPY-PtdCho and unsaturated 1,3 DAG. Incorporation of the SUVs into cell membranes in green, DGKε in red. EM images show 1,3 DAG completely rescued NE reformation. Images representative of n = 11 experiments. Scale bars: confocal 10 μm, CLEM as indicated on the images.

Depletion of DAG could not be quantified by transfecting the C1 domain in rapalogue- treated LBR/DGKε co-expressing cells, as DGKε and the C1 domain would compete for the same substrate. An alternative was to rescue the phenotype by delivery of DAG using small unilamellar vesicles (SUVs <50 nm diameter) composed of unsaturated DAG and PtdCho (20∶80 mole% 1,2 DAG:PtdCho) after the addition of rapalogue to LBR/DGKε co-expressing metaphase cells. Under these conditions, the NE reformed (Fig. 5B). The incorporation of 1,2 DAG:BODIPY-PtdCho SUVs was verified by fluorescence in interphase and mitotic cells (white arrows) (Fig. 5A).

Although rapalogue-treated cells progressed through cytokinesis, daughter cells did not go through a second division and died. When rescued by exogenous 1,2 DAG the NE was virtually normal and included nuclear pores [Fig. 5C, D and Movie S8] and cells were rescued from death. However, ER alterations persisted indicating a differential sensitivity of the newly formed multi-lamellar ER sheets and NE formation to DAG depletion.

To determine if the rescued NE phenotype was due to the added DAG acting as second messenger through C1 domain-containing proteins, we performed rescue experiments with the non-C1-binding DAG isomer 1,3 DAG [15]. Fig. 5E shows the incorporation of 1,3 DAG:BODIPY-PtdCho SUVs in interphase and mitotic cells (white arrows). NE phenotype was rescued both at 20∶80 and 40∶60 mole% (Fig. 5F-H). Fig. 5H images clearly show the rescued NE although the ER rescue was partial with fewer multi-lamellar sheets and more tubules, indicating that NE and ER rescues were differentially sensitive to DAG levels. All rescue experiments were single cell experiments performed 11 times with 90% reproducibility (Table 1).

To summarise, these data show that DAG is essential for the complete formation of the NE in mammalian cells and normal ER morphology. Importantly, 1,3 DAG rescue experiments emphasise a structural rather a signalling role for DAG.

### Interference with membrane fusion and aberrant ER architecture induced by DGK and Syn1 in live embryos and oocytes

**Figure 6:**
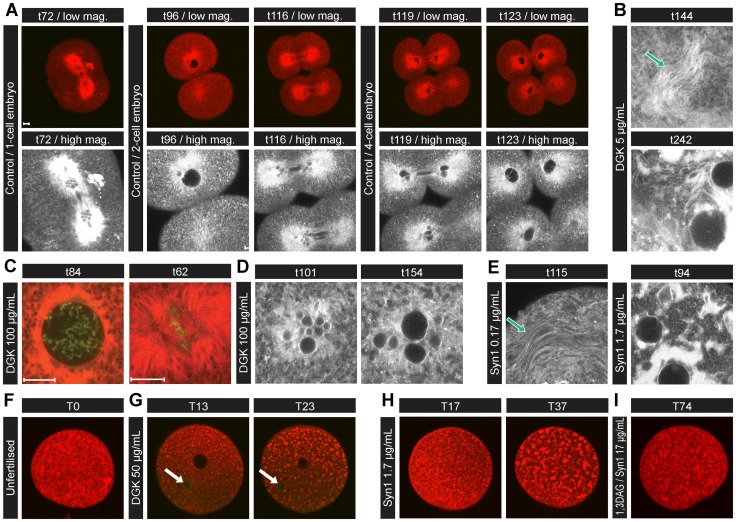
Effects of DGK and Synaptojanin1 microinjection on sea urchin embryos and eggs. (A) Fertilised eggs. The ER was labelled by microinjection of DiIC_18_ into sea urchin eggs between 10–25 min post fertilisation. Karyomeres in the first cell cycle resolve into individual nuclei between 72 and 96 min post-fertilisation and in the second cycle (4-cell stage) between 116 and 123 min. (B) At 5 μg/ml pipette concentration of DGK, curved stacked sheets of ER formed by 144 min that coalesced into more concentrated “aggregates”. (C) At 100 μg/ml of DGK, either chromosomes condensed but the NE did not break down or an apparently normal metaphase occurred but karyomere fusion (D) was greatly retarded compared to the first cycle controls. (E) Synaptojanin 1 (Syn1) induced a similar phenotype of karyomere resolution delay to DGK as well as formation of curved stacked sheets of ER that coalesced into “aggregates” of sheets (0.17 μg/ml and 1.7 μg/ml shown). Unfertilised haploid eggs (F) were injected with either 50 μg/ml DGK (G) or 1.7 μg/ml Syn1 (H) which rapidly converted the ER in a progressive coarsening of the pattern similar to fertilised eggs already detectable by 13 or 17 minutes post-injection (Movie S9). The upper dark circle in (G) images is the injected oil droplet; lower green circle (arrow) is the zygote nucleus with NE that does not undergo breakdown. (I) Unfertilised eggs were incubated with SUVs containing PtdCho and 1,3 DAG (80∶20 mole%) prior to Syn 1 injection (17 μg/ml) at T0. Their appearance did not change for more than 74 min (Movie S10). All embryos were injected with enzymes ∼40 min post-fertilisation. YOYO®-1 iodide was included to label nucleic acid green and monitor injection. Scale bars: 10 μm.

We next determined whether DAG affects the timing of nuclear membrane fusion as well as ER morphology in naturally synchronous echinoderm embryos, which are neither transformed nor differentiated. Since individual chromosomes with separate NEs (karyomeres) form during telophase of each cell cycle and resolve into a single large nucleus prior to each interphase by successive fusions of their outer and inner nuclear membranes (Fig. S5A) [34], karyomere resolution can be used as a readout of membrane fusion. To lower DAG by conversion to PtdOH or prevent its accumulation by depletion of its precursor PtdIns(4,5)P_2_, we microinjected serial dilutions of either DGK or the PtdIns(4,5)P_2_ and PtdIns(3,4,5)P_3_ 5-phosphatase synaptojanin 1 (Syn1) at 10 min prior to prophase (40 min post-fertilisation) into 1-cell echinoderm embryos whose ER had been labelled with DiIC_18_. Timing of karyomere fusion and cytokinesis of control embryos is shown in Fig. 6A.

At very low doses of DGK (0.05 μg/ml micropipette concentration), karyomere resolution and cytokinesis occurred in the first and second cycles on virtually the same schedule as controls. Typical extensive ER tubules were seen in the peripheral regions of cytoplasm not close to the chromosomes indicating a lack of artefact due to injection or manipulation of the embryos (Fig. S5B). Similar to controls, the ER was found in concentrated sheets near the chromosomes during mitosis, while peripheral cytoplasm contained ER tubules and a few dispersed sheets. However at 5 μg/ml DGK, the ER formed swirling sheet patterns throughout the cytoplasm at the expense of the fine tubules (Fig. 6B, t144, arrow), even in embryos able to undergo extensive karyomere resolution. These stacks later gave way to denser, more aggregated structures (Fig. 6B, t242). At 100 μg/ml DGK, either the NE did not break down, chromosomes condensing to prophase- or metaphase-like configurations within the NE and karyomeres failing to form, or an apparently normal metaphase occurred (Fig. 6C). The latter was followed by delayed formation and resolution of karyomeres at telophase (Fig. 6D). Resolution was incomplete even after controls had completed two cell cycles to reach the 4-cell stage. At 500 μg/ml DGK, embryos did not form karyomeres even if the NE broke down (Fig. S5C). They showed extensive reorganisation of the ER into large gently curved stacked sheets, which formed progressively from the fine tubular network that led to coarse “aggregated” ER patterns. This was sometimes followed by disorganised contractions or aberrant attempts at cytokinesis.

Embryos injected with Syn1 showed similar phenotypes to DGK. Low doses (0.017 μg/ml) slightly delayed karyomere resolution in the first cell cycle but after cytokinesis and NE breakdown, the embryos slowly progressed to the second metaphase (∼40 min later than controls), no further karyomeres formed and the cells did not divide again. Small “aggregates” of ER sheets appeared throughout the cytoplasm (Fig. S5D). At 0.17 μg/ml Syn1, embryos arrested at prophase of the first cell cycle, with no NE breakdown and an ER phenotype similar to the intermediate doses of DGK (as in Fig. 6B, t144), characterised by large curved sheets coexisting with smaller regions of tubules (Fig. 6E, t115) which eventually led to progressive coarsening of the ER pattern, the dominant form at higher doses (Fig. 6E, t94). Embryos then underwent disorganised contractions but without successful cytokinesis by the time controls had reached the 4-cell stage. At 1.7 μg/ml Syn1, NE breakdown did not occur and “coarsening” of the ER was more rapid (Fig. 6E, S4E). The curved sheets in appropriate orientations appeared as stacks (Fig. 6B, t144; 6E, t115) and therefore are likely precursors to the “aggregates” in which individual sheets cannot be resolved by light microscopy. The stacked sheets in the embryo may correspond to the multi-lamellar sheets noted in the HeLa cell experiments.

To eliminate cell cycle variables and evaluate how acutely the ER is altered, *unfertilised* oocytes were injected with DGK or Syn1. These metabolically quiescent cells have completed meiosis and are arrested in G1 until activation by sperm. Fig. 6F shows their fine pattern of ER sheets and tubules at T0 (pre-injection). By 13 min post-injection of DGK (T13) rearrangement of the ER to a coarse pattern could already be seen (Fig. 6G). This progressed for >30 min, strongly reminiscent of the progression seen in embryos. Chromatin of the egg nucleus remained dispersed for >120 min and the female NE never broke down (arrow), indicating the egg did not enter the cell cycle by parthenogenesis. Results with Syn1 in unfertilised eggs (Fig. 6H and Movie S9) were virtually identical. Finally, eggs were prevented from forming the altered ER phenotype by delivery of 1,3 DAG in SUVs prior to injection of Syn1 (Fig. 6I and Movie S10).

To summarise, in naturally synchronous echinoderm embryos, where levels of DGK or Syn1 have been titrated by microinjection, high doses led to loss of ER tubules and formation of aberrant stacked sheets or large curved sheets, as well as lack of NE breakdown and karyomere formation. At lower doses, karyomeres formed but fusion of their nuclear membranes was inhibited or greatly delayed. At still lower doses, karyomere resolution took place at virtually the same time as controls, even in cells showing alterations of ER. The same phenotypes appeared in unfertilised oocytes, thus ruling out a requirement for cell cycle progression or the specific signalling pathways associated with it. The echinoderm experiments did not introduce exogenous proteins such as LBR, nor transfection or synchronising drug treatments, yet led to rapid alterations of the ER membrane compartment similar to those observed in cultured mammalian cells.

The convergence of data derived from the different techniques and different cell types presented here indicates not only a critical role for DAG in membrane functions such as fusion, but also in morphology or organelle shaping, and demonstrates that these roles cannot be attributed solely to proteins. Theoretical studies of the bending and closure of flat, double membrane sheets into curved structures emphasize the relationship between their highly curved rim regions and their less curved sheets. Bending energy is dependent on the ratio of the radius of the flat sheet (r_sheet_) and of the rim (r_rim_) [19]. As sheets grow or their chemical composition changes, altering spontaneous curvature parameters, the energy required for bending or closure can be reduced until the achievement of a metastable state wherein small changes lead to shape alteration or fusion.

Our data suggest that lipids contribute to such changes. We previously showed that acutely depleting the phospholipids PtdIns3P and PtdIns(3,5)P_2_ (exhibiting spontaneous positive curvature) from early endosomes results in elongated tubules characterised by high cross-sectional membrane curvature [27]. In this paper, we show that depleting levels of DAG (exhibiting spontaneous negative curvature) leads to loss of NE assembly and formation of multi-lamellar sheets of ER at the expense of tubules. We suggest that the balance and asymmetry of lipids of positive and negative curvature affects the r_sheet/_r_rim_ *in vivo*.

We show that the aberrant NE phenotype is reversed by addition of SUVs containing unsaturated 1,3 DAG although the multi-lamellar sheets remain. In control telophase cells, numerous ER tubules extend to the nuclear membrane [35], whereas in cells depleted of DAG the multi-lamellar sheets have fewer tubular extensions connected to the NE. Previously, it was shown that ectopic expression of some proteins causes the ER to form small coils [36], [37]. We suggest that to form highly-curved ER tubules, sufficient DAG is required whereas depletion of DAG from ER membranes results in multi-lamellar sheets. To rescue the fragmented NE, additional proteins may not be required, as 1,3 DAG (the non-protein binding isomer of 1,2 DAG) completely reverses the phenotype, and that NE formation does not require a completely normal ER. Pre-treatment of unfertilised oocytes with 1,3 DAG also shows that DAG is needed for maintaining the ER.

The remarkable similarity of phenotypes in our experiments strongly suggests the conservation of a structural role of DAG across the deuterostome superphylum. Our results add two new conserved functions to diacylglycerol *in vivo*: a structural role in organelle shaping, and a role in localised extreme membrane curvature required for fusion for which proteins alone are an insufficient explanation.

## Materials and Methods

### Cells, antibodies and lipids

Human cervical cancer epithelial HeLa cells and African green monkey kidney fibroblast-like COS-7 cells were obtained from the American Type Culture Collection (ATCC; CCL-2 and CRL-1651 respectively). The anti-LBR antibody (Abcam) is a rabbit monoclonal (E398L), the anti-calreticulin antibody (Abcam) is a rabbit polyclonal. Lipids were purchased from Avanti Polar Lipids, PMA from Sigma, NBD-DAG from Cayman Chemical and BODIPY-PtdCho from Invitrogen.

### Cloning

PKCε LBR P58 ΔTM2-8, diacylglycerol kinase ε (DGKε) and pGEX-SKIP were kindly provided by Peter J. Parker (London Research Institute, UK), Susan Smith (New York University School of Medicine), Michael Wakelam (The Babraham Insitute, UK) and Rudiger Woscholski (Imperial College London, UK) respectively.

The original dimerisation constructs subsequently modified by Natali Fili were provided by Clontech iDimerize Inducible Heterodimer System [27]. RFP-FlagFRB-MTM2 and EGFP-2FKBP-Rab5 were as described previously [27].

### Constructs

SKIP (Skeletal muscle and Kidney enriched Inositol Phosphatase; phosphoinositide 5-phosphatase) was PCR amplified from pGEX-SKIP with flanking KpnI/BamHI restriction sites. The PCR product was subcloned into the pCR-Blunt II-Topo vector (Invitrogen #45-0245) and two internal BamHI sites were removed by site-directed mutagenesis. Topo-SKIP and RFP-Flag-FRB-MTM2 were digested with KpnI and BamHI and fragments corresponding to SKIP (insert) and RFP-Flag-FRB (vector) were purified and ligated to give the final construct RFP-Flag-FRB-SKIP. The SKIP D310G mutant was created by site-directed mutagenesis using the oligos: Sense–cgtacggcatcagcgGccacaagcctgtctcc and Antisense–ggagacaggcttgtggCcgctgatgccgtacg.

EGFP-PKCε was made by removing an internal BamHI site using the oligos: Sense–gccccacaagttcggcatccacaactacaaggtccccacg and Antisense–cgtggggaccttgtagttgtggatgccgaacttgtggggc. PKCεC1aC1b was PCR amplified using oligos: Sense–agggatccatgcacaacttcatggccacctacttgcggcaac and Antisense–ttgccgcaagtaggtggccatgaagttgtgcatggatccct. The PKCε W264G mutant was created by site-directed mutagenesis using the oligos: Sense–cactgtggctccctgctctacggcctcttgcggcagggc and Antisense–gccctgccgcaagaggccgtagagcagggagccacagtg.

To create EGFP-2FKBP-LBRΔTM2-8, an EcoRI site was inserted into the EGFP-2FKBP vector. LBRΔTM2-8 was PCR amplified with flanking EcoRI/BamHI sites and then subcloned into EGFP-2FKBP digested with EcoRI and BamHI. The dark LBR construct was created by site-directed mutagenesis using the oligos: Sense–cgtgaccaccctgaccCTcggcgtgcagtgcttc and Antisense–gaagcactgcacgccgAGggtcagggtggtcacg.

RFP-Flag-FRB-DGKεK was made by PCR amplifying DGKεK with flanking KpnI sites and subcloning into KpnI-digested RFP-Flag-FRB-MTM2. The kinase-inactive DGKεK D434N mutant was created by site-directed mutagenesis using oligos: Sense–gtttgtggagggaatgggactgtagggtggg and Antisense–cccaccctacagtcccattccctcccacaaac.

### Cell Culture, transfections, membrane labelling and immunofluorescence

HeLa cells were maintained in DMEM supplemented with 10% fetal bovine serum (HeLa) or donor calf serum (COS-7) and seeded at 200,000 per well of a 6 well plate or in a MatTek dish.

Cells were transfected with 0.5 μg DNA of each construct using Lipofectamine LTX and PLUS reagent (Invitrogen) in OPTIMEM medium (Gibco BRL) as recommended by the manufacturer. The cells were left for 4 hours in the transfection mix before replacing the medium with antibiotic-free medium. Experiments were performed 24–36 hours after transfection.

DiOC_6_ (3,3′-dihexyloxacarbocyanine iodide; 5 μM) was added to the media and incubated for 5 minutes at 37°C. ER Tracker (green; Molecular Probes) was used at a concentration of 1 μM, added to the media and incubated for 30 min at 37°C. For the phorbol 12-myristate 13-acetate (PMA) assay, EGFP-PKCεC1a-C1b-transfected cells were incubated in a 5% humidified chamber with 10% CO_2_ adapted to a Nikon Low Light Imaging System. PMA was added to the cells to 800 nM and incubated 20 min before acquisition of the images. For immunofluorescence, cells were washed once with PBS and fixed with 4% (w/v) paraformaldehyde (PFA) at 22°C for 10 minutes. Excess PFA was removed by further washes with PBS. Cells were permeabilised and blocked using 0.5% (w/v) Saponin and 3% (w/v) BSA. Primary anti-LBR or anti-calreticulin antibodies or the recombinant PLCδ1 PH domain were added for 1 h. After three washes in PBS, the Alexa Fluor 647-conjugated secondary antibody (LBR) or DyLight 649-conjugated antibody (calreticulin) was added for an hour. Cells were then stained 5 minutes with Hoechst 33343 (2 μg/mL) and mounted in ProLong Gold anti-fade reagent (Invitrogen).

### Rapalogue Dimerisation Device and Confocal Imaging

Cells were incubated in a 5% humidified chamber with 10% CO_2_ adapted to a 710 LSM Zeiss confocal microscope. A series of images was acquired prior to the addition of the rapalogue (A/C heterodimiser – Clontech) to the medium at a final concentration of 500 nM. Translocation of DAG kinase to the NE and ER (LBR localisation) occurred from 30 minutes after rapalogue addition. The images were initially acquired every 20 minutes at low resolution (512×512 with a 0.6 zoom) from interphase to late cytokinesis. A further series of experiments was performed at high resolution (1024×1024 with a zoom of 2) from early anaphase to late cytokinesis. Once the rapalogue was added the heterodimerisation was sustained for 8–12 hours. Each experiment was performed at least three times. All images were treated the same manner; i.e only minor adjustments of brightness and contrast were applied to every pixel.

### PtdCho/DAG Rescue Experiments

100 μL of 1 mM PtdCho:unsaturated DAG (80∶20 or 60∶40 mole %) stock solution in PBS was prepared, sonicated (3 cycles of 10sec) and 20 μL (10 μM final) added to the culture medium at least 1h after addition of rapalogue on cells at metaphase. For experiments with Egg-PtdCho:DAG SUVs (1,2- or 1,3-DAG – Fig. 4A and Fig. 4E), β-BODIPY 500/510 C12-DAG SUVs (containing the same DAG in the same amount) were prepared in parallel and incorporation into the cells was verified with BODIPY-PtdCho:DAG SUVs prior to each experiment. For experiments with β-BODIPY 500/510 C12-1,3 DAG SUVs (60∶40 mole % – Fig. 4E), incorporation into the cell membranes was visible from the addition of SUVs at metaphase. Care was taken for the 1,3 DAG experiments not to be in acidic conditions. All SUVs were made just prior to every experiment. Incorporation to the cell membranes was visible from the addition of SUVs to the medium.

### Quantification of NE phenotype in single cell experiments of HeLa cells co-expressing LBR and DGKε

Cells were selected on their levels of EGFP-2FKBP-LBRΔTM2–8 (LBR) and RFP-Flag-FRB-DGKεK (DGKε). Cells expressing low levels of LBR or DGKε, that is with a low S/N, or in the wrong plane, were excluded. The NE phenotype at cytokinesis (complete *versus* fragmented) was evaluated by the presence or absence of a complete NE in all z-planes.

### ER-enriched Nuclei Isolation

HeLa cells were grown on 500 cm^2^ tissue culture dishes in Dulbecco's Modified Eagle's Medium (DMEM) (Invitrogen #31966). After harvesting by trypsinisation and washing twice in ice cold phosphate buffered saline (PBS), cells were incubated on ice for 1 hour in swelling buffer (10 mM Hepes, pH 7.9, 10 mM KCl, 1.5 mM MgCl_2_, 0.5 mM DTT). Cells were lysed by nitrogen cavitation in a cell disruption bomb (Parr Instrument Company, Illinois USA). Samples were incubated on ice at a pressure of 200 psi for 5 minutes before decompression and sample collection. Samples were centrifuged at 218 g for 5 minutes to pellet the nuclei (the supernatant was discarded). Nuclei were resuspended in solution S1 (0.25 M Sucrose, 10 mM MgCl_2_), layered over an equal volume of solution S2 (0.35 M Sucrose, 0.5 mM MgCl_2_) and centrifuged at 1430 g for 5 minutes. After discarding the supernatant, the pellet containing nuclei and some whole cells was resuspended in 200 μl Tris buffered saline (TBS). 10 μl of this was settled onto a poly-L-lysine coated coverslip (BD Scientific) and stained with DiOC_6_ (2 μM) and Hoechst 33343 (2 μg/ml) to assess the efficiency of nuclei isolation. Lipids were extracted from the remaining 190 μl.

### Lipid extraction and DAG analysis

Lipids were extracted from nuclei samples using a modified Folch extraction, described in detail here [38]. Nuclei were added to acidified chloroform:methanol (2.5∶1), sonicated and filtered. After addition of 0.2 volumes K_4_EDTA (0.2 M, pH 6), samples were centrifuged at 680 g. The lower phase was retained and dried down completely under nitrogen. Lipids were resuspended in chloroform:methanol (1∶1) and internal standards added as follows – 12:0/12:0 DAG 67 ng, 12:0/12:0 PtdOH 33 ng, 12:0/12:0 PtdGly 33 ng, 12:0/12:0 PtdCho 333ng, 12:0/12:0 PtdEth 267 ng, 12:0/12:0 PtdSer 267 ng, 17:0/20∶4 PtdIns 327 ng.

DAG analysis was performed on a Shimadzu IT-TOF LC/MS/MS system hyphenated with a five-channel online degasser, four-pump, column oven and autosampler with cooler (Prominence HPLC, Shimadzu). Lipid classes were first separated on a normal phase silica column (2.1×150mm, 4 μm internal diameter, MicroSolv Technology) using a solvent gradient from hexane/chloroform (3∶1) to dichloromethane/chloroform/acetonitrile/water/ethylamine (30∶30∶30:10∶0.12). To identify and quantify molecular species we used accurate mass (mass accuracy 5 ppm) and tandem MS, as well as comparison with appropriate lipid standards. IT-TOF mass spectrometer operation conditions: ESI interface voltage +4.5kv for positive ESI and -4kv for negative ESI, heat block temperature 230 C, nebulising gas flow 1.4L/min, CDL temperature 210 C, drying gas pressure 100 psi. All solvents used for lipid extraction and LC/MS/MS analysis are LC-MS grade from Fisher Scientific.

### Correlative Light and Electron Microscopy (CLEM)

Cells were grown on gridded glass coverslips in MatTek dishes. Live cells were treated and followed to the required stage of mitosis using confocal microscopy. Cells were then fixed in 4% paraformaldehyde in 0.1 M phosphate buffer (PB) pH 7.4 to halt the cell cycle prior to re-imaging for brightfield and high magnification fluorescence signals. Secondary fixation was performed in 1.5% glutaraldehyde/2% paraformaldehyde in 0.1 M PB for 30–60 minutes. After fixation, coverslips were carefully removed from the MatTek dishes and washed several times in 0.1 M PB. The cells were post-fixed in 1.5% potassium ferricyanide/1% osmium tetroxide for one hour, before rinsing in PB, and incubating in 1% tannic acid in 0.05 M PB for 45 minutes to enhance membrane contrast. After a brief rinse in 1% sodium sulphate in 0.05 M PB, the coverslips were washed twice in distilled water, dehydrated through an ascending series of ethanol to 100% prior to infiltration with Epoxy resin and polymerisation overnight at 60^o^C. The coverslips were removed from the resin blocks by plunging briefly into liquid nitrogen. The cells of interest were identified by correlating the grid and cell pattern on the surface of the block with previously acquired confocal images. The area of interest was cut from the block and further trimmed by hand using a single edged razor blade to form a small trapezoid block face for serial ultrathin sectioning. Using a diamond knife, serial ultrathin sections of 70 nm thicknesses were cut through the entire extent of the cells of interest (∼80 to 140 sections) and collected on 1.5% formvar-coated single slot grids. The sections were counterstained with lead citrate to further enhance contrast prior to viewing in the electron microscope (FEI Tecnai G^2^ Spirit BioTWIN with Gatan Orius CCD camera). Serial images were stacked and aligned, and the NE, ER and centrioles were manually segmented using Amira (Visage Imaging, Berlin). For the purpose of segmentation, membranes contacting chromatin were assigned as NE. ER was segmented to a distance of approximately 3 μm from the NE in the control anaphase, control telophase and low DGKε cells. Large ER coils were additionally segmented in the high DGKε cells. Due to the size of the datasets, segmentations of the ER were binned 2,2,1 (x, y, z) prior to creating surfaces, whereas the NE (and centriole) surfaces were created without binning to minimise appearance of artefactual gaps in the 3D rendered model. Movies were created from 2D tiff stacks using Quicktime Player 7 Pro, and compressed using Stomp (Shinywhitebox Ltd).

### Microinjection of DGK and Syn1 into echinoderm embryos and oocytes

Echinoderm oocytes (*L. pictus*) were fertilised at room temperature in Millipore filtered (0.45μm) artificial sea water (MFSW) containing 3 mM 3-amino-1,2,4-triazole (ATA) to soften fertilisation membranes, washed at 5 min post-fertilisation (pf) in MFSW without ATA and loaded by capillary action into chambers constructed with coverslips and double sided tape spacers [39]. Between 10–25 min pf, embryos were manually microinjected with 2–8 pl of a saturated solution of DiIC_18_ (1,1′-dioctadecyl-3,3,3′,3′-tetramethylindocarbocyanine perchlorate, Sigma-Aldrich 42364) in Wesson oil which fluorescently labels the endoplasmic reticulum red [40]. At 40 min pf, 5–20 pl of sn-1,2-diacylglycerol kinase (DGK; Sigma Aldrich #D3065) or the recombinant 5-phosphatase Synaptojanin 1 (Syn1) (1/200 to 1/20,000 dilution of 0.33 mg/ml stock in LB) (Syn1 prepared in Larijani lab) at various concentrations in LB (10 mM HEPES pH 8.0, 250 mM NaCl, 25 mM EGTA, 5 mM MgCl_2_, 110 mM glycine, 250 mM glycerol, 1 mM DTT, and 1 mM PMSF) was injected. DGK and Syn 1 were also injected into unfertilized eggs. Microinjections were performed according to procedures of Jaffe and Terasaki (http://mterasaki.us/panda/injection). Some eggs were co-injected with enzymes and 100 μM YOYO®-1 iodide (Invitrogen) to fluorescently label nucleic acids green. After sealing the open end of the chambers with mineral oil, further embryo culture and observations were by confocal microscopy (Zeiss Pascal software, Zeiss Axiovert 200 microscope, 40X objective) performed at room temperature.

### PtdCho/DAG Sea Urchin Rescue Experiments

SUVs for sea urchin eggs were made in LB buffer [41] as above and diluted 10-fold into a suspension of eggs in sea water and incubated for 30 min at room temperature. Eggs were washed in sea water to remove unincorporated SUVs and microinjected.

## Supporting Information

Figure S1 Localisation of diacylglycerol with PtdCho/NBD-DAG SUVs in mammalian cells at interphase and response of DAG probe to phorbol ester. (A) HeLa cells transfected with EGFP-PKCεC1aC1b (left panel) or labelled by addition of small unilamellar vesicles (SUVs) composed of polyunsaturated PtdCho and unsaturated NBD-DAG (60/40 mole% respectively) to the medium (right panel) and imaged by live confocal microscopy. In both conditions, DAG was localised at the nuclear envelope (NE) (yellow arrows), ER (green arrow) and Golgi (white arrows). (B) HeLa cells were transfected with EGFP-PKCεC1aC1b (left panel) or its DAG non-binding mutant (C1b W264G – right panel) and imaged by video microscopy after 800 nM PMA treatment for 20 minutes. EGFP-PKCεC1aC1b translocated to the plasma membrane (red arrows) but the W264G mutant did not respond to PMA and therefore did not translocate to the plasma membrane. Scale bars: 10 μm.
(TIF)

Figure S2 LBR localisation in HeLa cells and molecular composition of DAG in isolated nuclei by lipid mass spectrometry. (A) Localisation of endogenous LBR (green), detected by indirect immunofluorescence. To label chromatin, cells were incubated with Hoechst 333432 (red). (B) HeLa cells transfected with EGFP-LBR and EGFP-2FKBP-LBRΔTM2-8 showed the same NE and ER localisation. (C) Confocal images of whole HeLa cells and isolated nuclei enriched in ER and NE. Membranes were labelled with DiOC6 (green), chromatin with Hoechst 333432 (red). DAG composition by mass spectrometry showed that the ER and NE were enriched in unsaturated DAG species. Scale bars: 10 μm.
(TIF)

Figure S3 Endogenous PtdIns(4,5)P_2_ localisation detected by recombinant PLCδ1 PH domain. (A) The transfected EGFP-PLCδ1 PH domain detected PtdIns(4,5)P_2_ at the plasma membrane of interphase HeLa cells (left panel) whereas the recombinant PLCδ1 PH domain (right panel) detected PtdIns(4,5)P_2_ at the perinuclear and nuclear envelope regions (green and yellow arrows respectively). (B-C) CLEM images of HeLa cells fixed at cytokinesis and permeabilised by 0.5% Saponin (C). Control cells were not permeabilised (B). The morphology of the endomembranes was unaffected by 0.5% Saponin. Membranes were labelled with DiOC6 (green). Scale bars: fluorescence 10 μm; CLEM as indicated on the images.
(TIF)

Figure S4 Correlative Light and Electron Microscopy (CLEM) workflow. Cells were grown on gridded glass coverslips. Cells of interest were identified and imaged using live confocal microscopy and fixed at the required stage of mitosis. Their position was mapped with respect to the grid using bright-field light microscopy. The coverslip was then stained, dehydrated and resin-embedded, at which point the grid is imprinted on the surface of the resin block. The cell was relocated, and 80–140 serial sections were collected through the entire cell. Sections were imaged in order, aligned using Amira software, and the features of interest were selected (segmented) to produce a 3D model. NE (red), endoplasmic reticulum (blue), centriole (yellow). A telophase cell is shown as an example of the process.
(TIF)

Figure S5 Effects of DGK and Synaptojanin 1 microinjection on sea urchin embryos and eggs. (A) Diagrammatic representation of two successive membrane fusions undergone by karyomeres based on electron microscopy data of Longo (Longo, 1972). (B) Embryos injected with 0.05 μg/ml DGK. Timing of karyomere fusion is virtually identical to controls (see Fig. 6A). (C) Embryos injected with 500 μg/ml DGK. NE breakdown is delayed, karyomeres do not form and ER coarsens with loss of tubules and forms large curved sheet structures. (D) Embryos injected with 0.017 μg/ml Syn1. Timing of karyomere fusion is virtually identical to controls but embryos arrest at 2-cell stage. (E) Embryos injected with 1.7 μg/ml Syn1. Extensive coarsening of ER with loss of tubules. ER was labelled by microinjection of DiIC18 into sea urchin eggs between 10–25 min post-fertilisation. Enzymes were injected ∼40 min post-fertilisation. YOYO®-1 iodide was included to label nucleic acid green and monitor injection. Scale bars: 10 μm.
(TIF)

Movie S1 The distribution of DAG in an EGFP-PKCεC1aC1b-transfected HeLa cell imaged every minute by live confocal microscopy from early anaphase to post-cytokinesis. ER tubules, NE reformation from the rim and reformation of the Golgi can be observed. All z sections are shown at each time point.
(MOV)

Movie S2 LBR localisation in the absence of rapalogue in an EGFP-2FKBP-LBRΔTM2-8 (LBR)/RFP-Flag-FRB-DGKεK (DGK)-transfected HeLa cell imaged every minute by live confocal microscopy from early anaphase to post-cytokinesis. A complete NE formed between late telophase and cytokinesis. All z sections are shown at each time point.
(MOV)

Movie S3 LBR localisation in the presence of rapalogue in an EGFP-2FKBP-LBRΔTM2-8 (LBR)/RFP-Flag-FRB-DGKεK (DGK)-transfected HeLa cell imaged every minute by live confocal microscopy from early anaphase to post-cytokinesis. NE reformation, impaired, had not occurred at cytokinesis. All z sections are shown at each time point.
(MOV)

Movie S4 Correlative light and electron microscopy (CLEM) images of aligned serial ultrathin sections of 70 nm thickness of a control HeLa cell, at telophase. The NE was close to completion and the ER a combination of sheets and tubules.
(MOV)

Movie S5 CLEM images of aligned serial ultrathin sections of 70 nm thickness of a rapalogue-treated, LBR/low DGK-expressing HeLa cell at telophase. The NE resembled that of an anaphase, with large gaps. The ER was partially tubular with some small multi-layered sheets.
(MOV)

Movie S6 CLEM images of aligned serial ultrathin sections of 70 nm thickness of a rapalogue-treated, LBR/high DGK-expressing HeLa cell at cytokinesis. The NE was highly fragmented and the ER morphology extremely aberrant, consisting in coils of multi-layered curved sheets.
(MOV)

Movie S7 3D models reconstructed from manually-segmented CLEM images of 70 nm sections of 1) control anaphase and 2) telophase, 3) rapalogue-treated, LBR/low DGK- and 4) high DGK-expressing HeLa cells. Dose-dependent effects upon DAG depletion include large gaps in the NE (red) and aggregation of the ER (turquoise).
(MOV)

Movie S8 CLEM images of aligned serial ultrathin sections of 70 nm thickness of a LBR and DGK transfected HeLa cell, upon rapalogue and addition of SUVs composed of Egg-PtdCho and polyunsaturated DAG (80/20 mole % respectively) at cytokinesis. The NE reformed, resembling control interphase cells.
(MOV)

Movie S9 The progressive alterations in ER architecture in echinoderm eggs injected with 17 μg/ml Syn1. The successive time points captured by confocal microscopy every 2 min from T = 17–57 post-injection.
(MOV)

Movie S10 Stable ER architecture in echinoderm eggs pre-treated with 1,3 DAG-containing SUVs prior to microinjection with 17 μg/ml Syn1. The successive time points captured by confocal microscopy every 2 min 13–53 min post-injection.
(MOV)
