# Supplementary figures and images for: Acute Manipulation of Diacylglycerol Reveals Roles in Nuclear Envelope Assembly & Endoplasmic Reticulum Morphology
Source: PLoS One. 2012 Dec 5;7(12):e51150. doi: 10.1371/journal.pone.0051150 (PMC3515572; doi:10.1371/journal.pone.0051150)

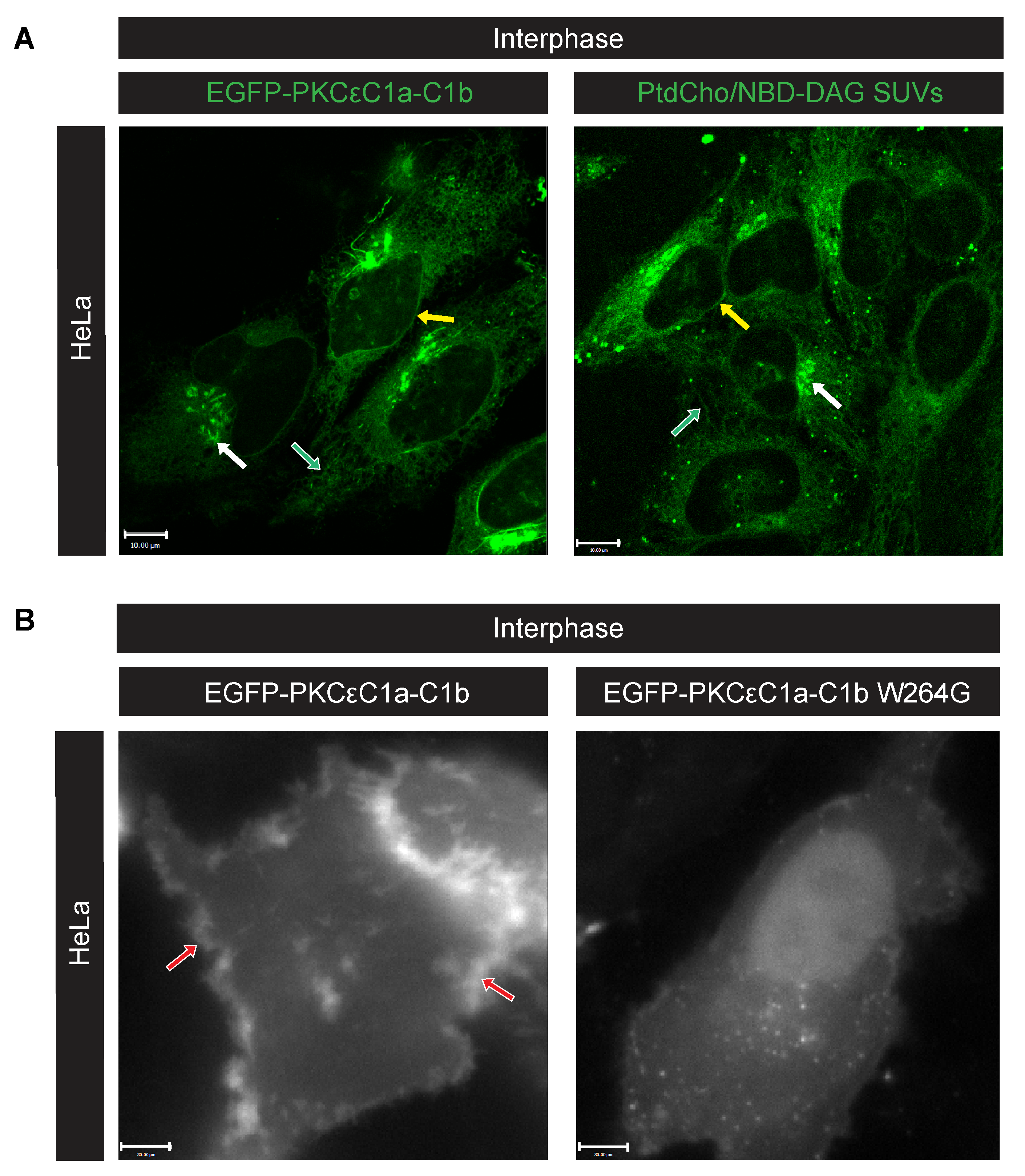

Supplement: Figure S1 — Localisation of diacylglycerol with PtdCho/NBD-DAG SUVs in mammalian cells at interphase and response of DAG probe to phorbol ester. (A) HeLa cells transfected with EGFP-PKCεC1aC1b (left panel) or labelled by addition of small unilamellar vesicles (SUVs) composed of polyunsaturated PtdCho and unsaturated NBD-DAG (60/40 mole% respectively) to the medium (right panel) and imaged by live confocal microscopy. In both conditions, DAG was localised at the nuclear envelope (NE) (yellow arrows), ER (green arrow) and Golgi (white arrows). (B) HeLa cells were transfected with EGFP-PKCεC1aC1b (left panel) or its DAG non-binding mutant (C1b W264G – right panel) and imaged by video microscopy after 800 nM PMA treatment for 20 minutes. EGFP-PKCεC1aC1b translocated to the plasma membrane (red arrows) but the W264G mutant did not respond to PMA and therefore did not translocate to the plasma membrane. Scale bars: 10 μm. (TIF) [file pone.0051150.s001.tif]

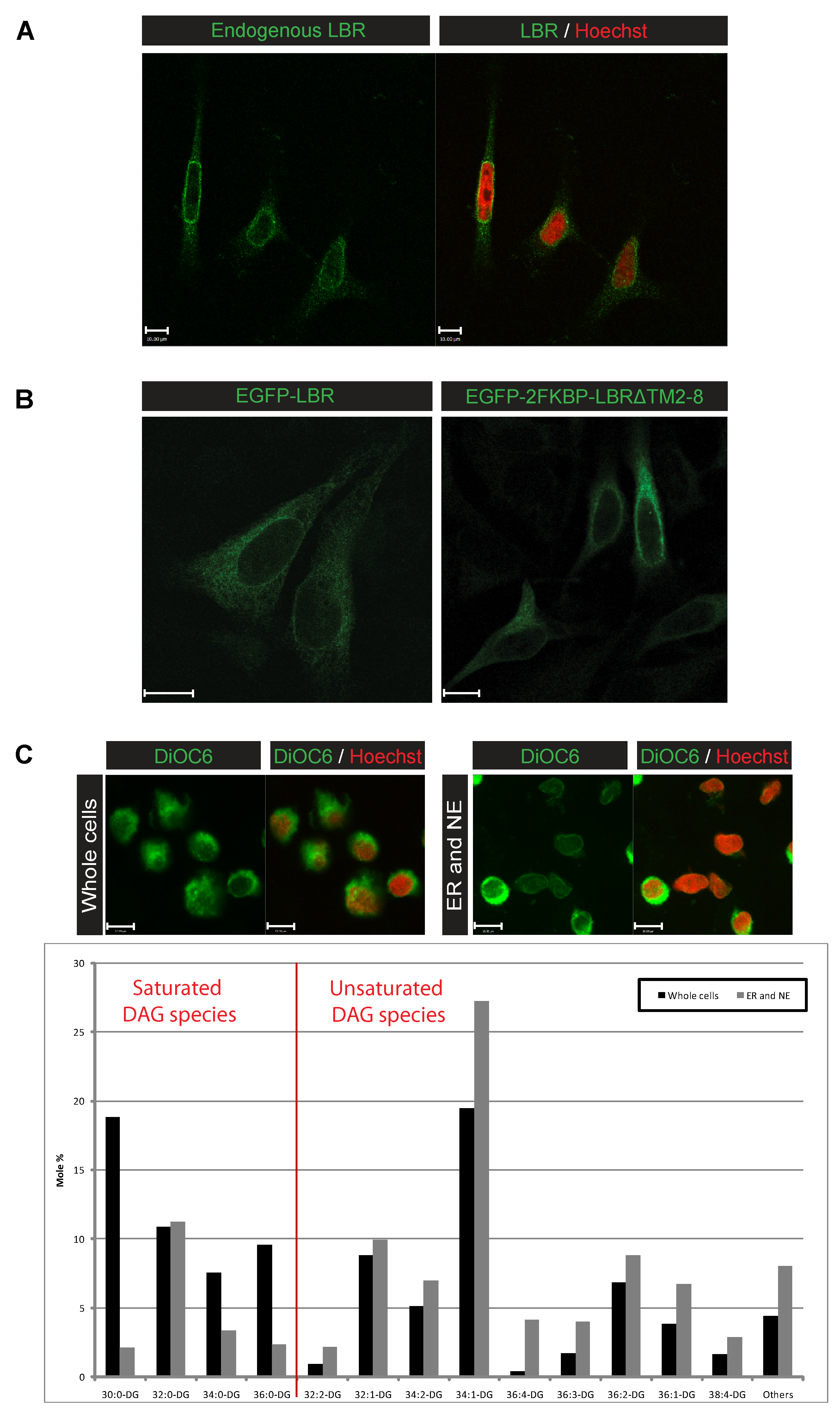

Supplement: Figure S2 — LBR localisation in HeLa cells and molecular composition of DAG in isolated nuclei by lipid mass spectrometry. (A) Localisation of endogenous LBR (green), detected by indirect immunofluorescence. To label chromatin, cells were incubated with Hoechst 333432 (red). (B) HeLa cells transfected with EGFP-LBR and EGFP-2FKBP-LBRΔTM2-8 showed the same NE and ER localisation. (C) Confocal images of whole HeLa cells and isolated nuclei enriched in ER and NE. Membranes were labelled with DiOC6 (green), chromatin with Hoechst 333432 (red). DAG composition by mass spectrometry showed that the ER and NE were enriched in unsaturated DAG species. Scale bars: 10 μm. (TIF) [file pone.0051150.s002.tif]

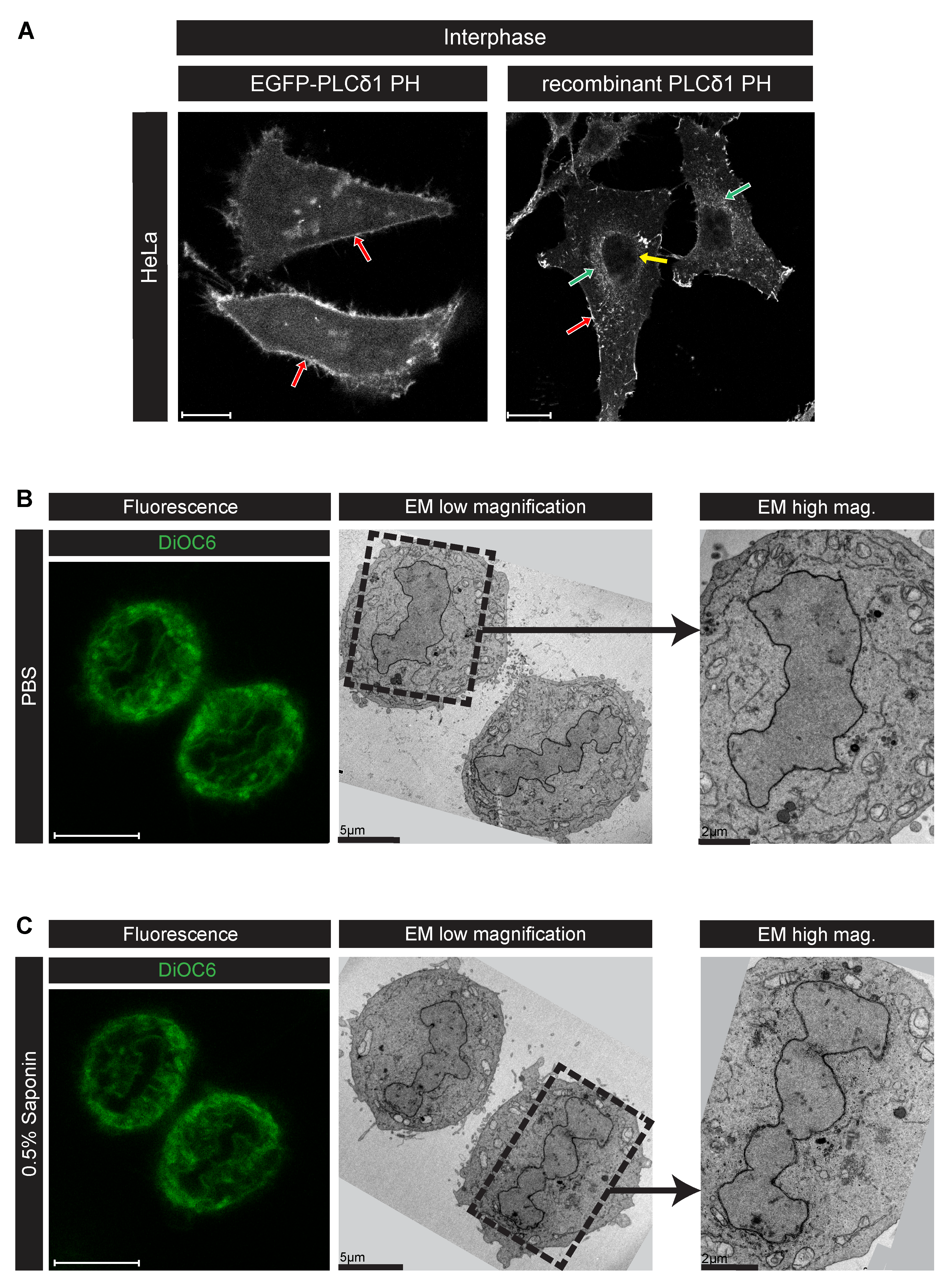

Supplement: Figure S3 — Endogenous PtdIns(4,5)P2 localisation detected by recombinant PLCδ1 PH domain. (A) The transfected EGFP-PLCδ1 PH domain detected PtdIns(4,5)P2 at the plasma membrane of interphase HeLa cells (left panel) whereas the recombinant PLCδ1 PH domain (right panel) detected PtdIns(4,5)P2 at the perinuclear and nuclear envelope regions (green and yellow arrows respectively). (B-C) CLEM images of HeLa cells fixed at cytokinesis and permeabilised by 0.5% Saponin (C). Control cells were not permeabilised (B). The morphology of the endomembranes was unaffected by 0.5% Saponin. Membranes were labelled with DiOC6 (green). Scale bars: fluorescence 10 μm; CLEM as indicated on the images. (TIF) [file pone.0051150.s003.tif]

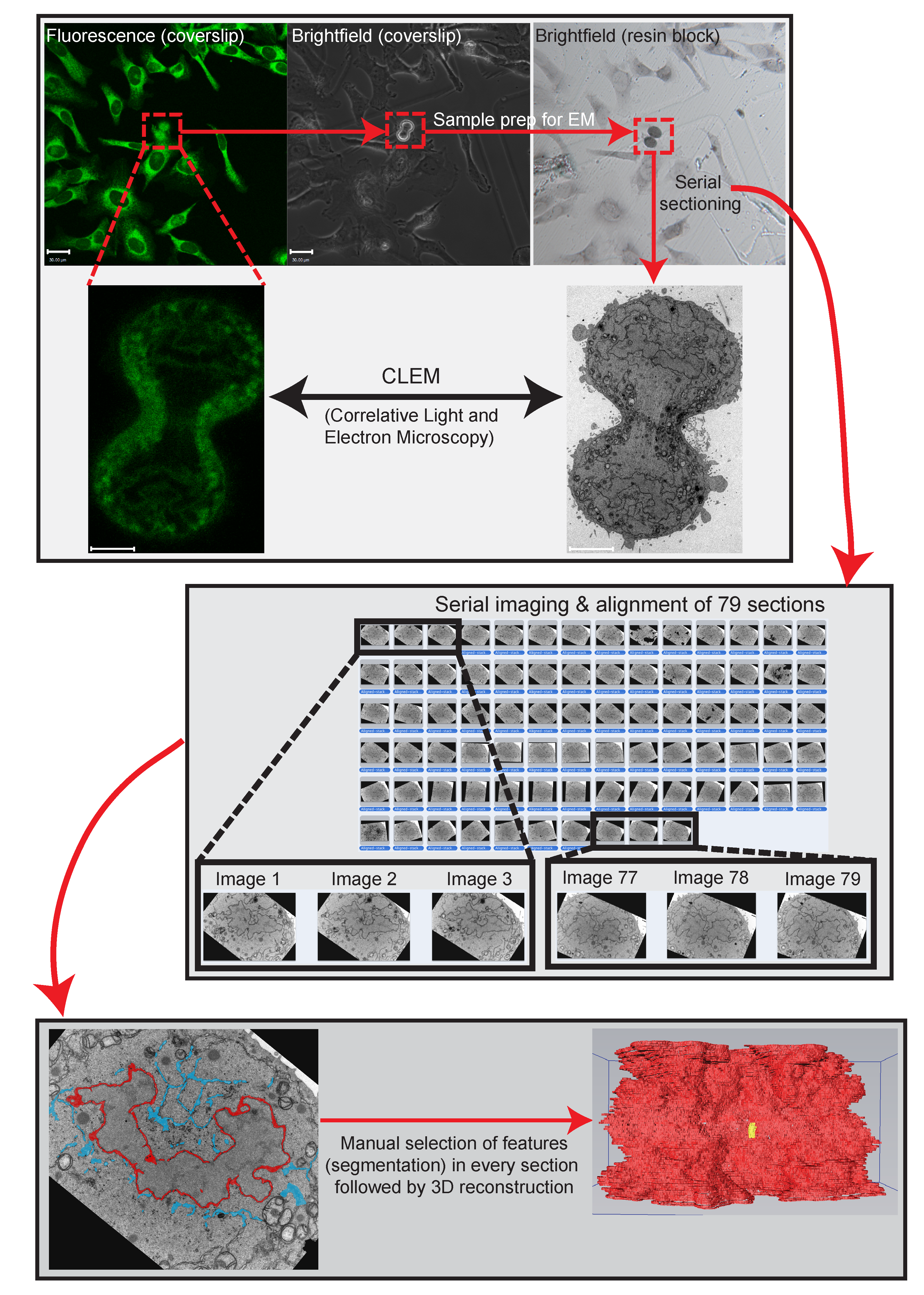

Supplement: Figure S4 — Correlative Light and Electron Microscopy (CLEM) workflow. Cells were grown on gridded glass coverslips. Cells of interest were identified and imaged using live confocal microscopy and fixed at the required stage of mitosis. Their position was mapped with respect to the grid using bright-field light microscopy. The coverslip was then stained, dehydrated and resin-embedded, at which point the grid is imprinted on the surface of the resin block. The cell was relocated, and 80–140 serial sections were collected through the entire cell. Sections were imaged in order, aligned using Amira software, and the features of interest were selected (segmented) to produce a 3D model. NE (red), endoplasmic reticulum (blue), centriole (yellow). A telophase cell is shown as an example of the process. (TIF) [file pone.0051150.s004.tif]

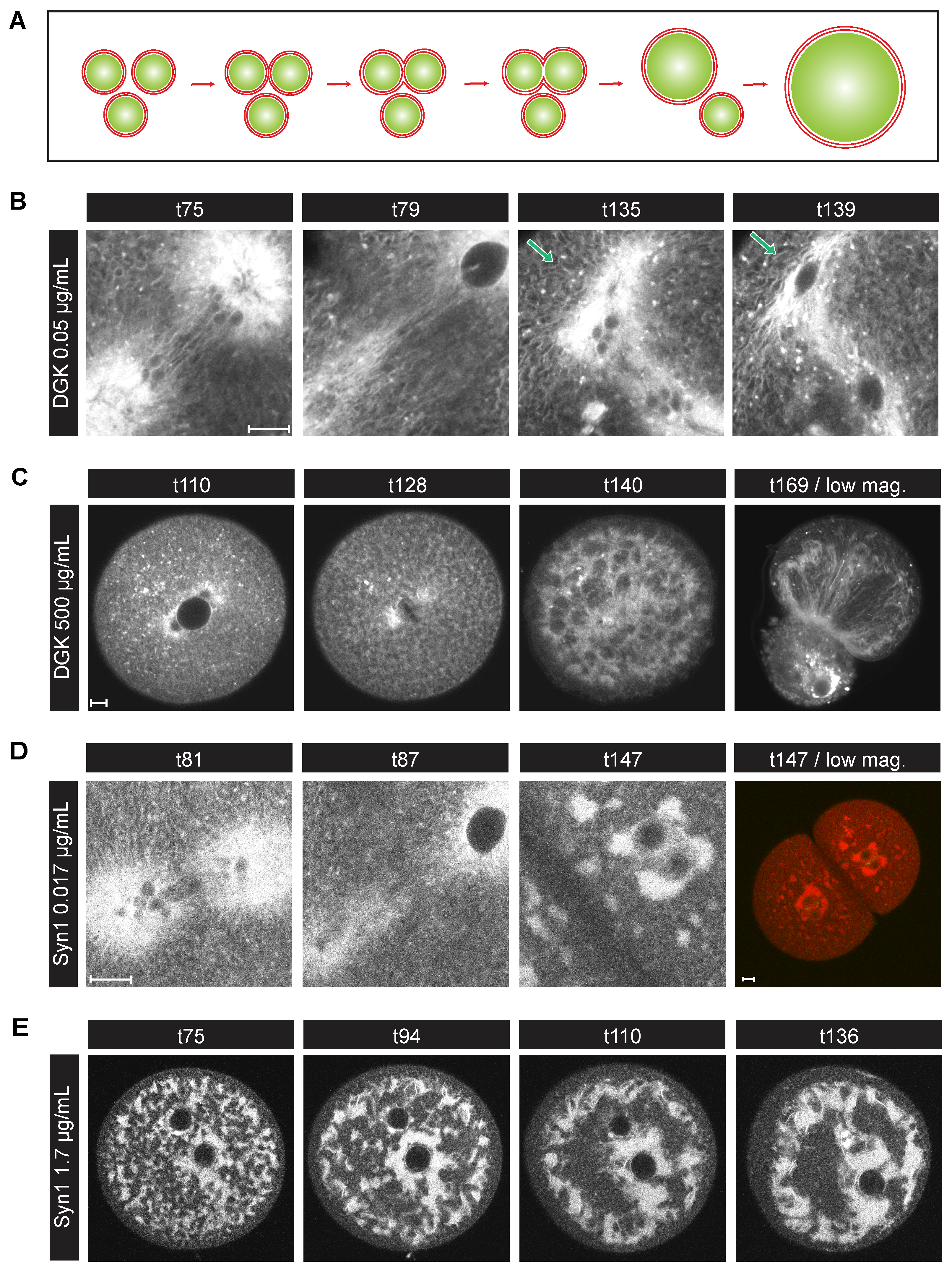

Supplement: Figure S5 — Effects of DGK and Synaptojanin 1 microinjection on sea urchin embryos and eggs. (A) Diagrammatic representation of two successive membrane fusions undergone by karyomeres based on electron microscopy data of Longo (Longo, 1972). (B) Embryos injected with 0.05 μg/ml DGK. Timing of karyomere fusion is virtually identical to controls (see Fig. 6A). (C) Embryos injected with 500 μg/ml DGK. NE breakdown is delayed, karyomeres do not form and ER coarsens with loss of tubules and forms large curved sheet structures. (D) Embryos injected with 0.017 μg/ml Syn1. Timing of karyomere fusion is virtually identical to controls but embryos arrest at 2-cell stage. (E) Embryos injected with 1.7 μg/ml Syn1. Extensive coarsening of ER with loss of tubules. ER was labelled by microinjection of DiIC18 into sea urchin eggs between 10–25 min post-fertilisation. Enzymes were injected ∼40 min post-fertilisation. YOYO®-1 iodide was included to label nucleic acid green and monitor injection. Scale bars: 10 μm. (TIF) [file pone.0051150.s005.tif]
